# Challenges and opportunities for conducting pre-hospital trauma trials: a behavioural investigation

**DOI:** 10.1186/s13063-023-07184-5

**Published:** 2023-03-02

**Authors:** Louisa Lawrie, Eilidh M. Duncan, Robert Lendrum, Victoria Lebrec, Katie Gillies

**Affiliations:** 1 Health Services Research Unit, 3Rd Floor Health Sciences Building, Institute of Applied Health Sciences, School of Medicine, Medical Sciences and Nutrition, University of Aberdeen, Foresterhill, Aberdeen, AB25 2ZD UK; 2 Barts Health NHS Trust, Royal London Hospital, St. Bartholomew’s Hospital, London, England

**Keywords:** Trials methodology, Feasibility, Behavioural Science, Pre-hospital

## Abstract

**Background:**

Trials in pre-hospital trauma care are relatively uncommon. There are logistical and methodological challenges related to designing and delivering trials in this setting. Previous studies have assessed challenges reported in individual trials rather than across the pre-hospital trial landscape to identify over-arching factors. The aim of this study was to investigate the challenges and opportunities related to the set-up, design and conduct of pre-hospital trauma trials from across the pre-hospital trial landscape and a specific pre-hospital trauma feasibility study.

**Methods:**

Semi-structured interviews were conducted with two cohorts of participants: research personnel who had experience of pre-hospital trials, either through direct involvement in conduct or through strategic oversight of national initiatives (*n* = 7), and clinical staff (*n* = 16) involved in recruitment to a pre-hospital trauma feasibility study. Thematic analyses were used to assess the barriers and enablers of conducting pre-hospital trauma trials. Two frameworks (The Capability Opportunity Motivation—Behaviour and the Theoretical Domains Framework) were used to guide analyses.

**Results:**

The barriers and enablers reported were relevant to several TDF domains and COM-B components. Across both cohorts, challenges associated with opportunities were reported and included the lack of research experience amongst pre-hospital staff, team dynamics within a rotating shift schedule, and the involvement of external organisations with diverse institutional priorities and infrastructures (e.g. Air Ambulances). The infrequency of eligible cases was also reported to affect the trial design, set-up, and conduct. Other barriers reported related to clinical equipoise amongst staff and institutional pressures, which affected motivation.

**Conclusions:**

This study has highlighted that pre-hospital trials face many context-specific but also generic challenges. Pre-hospital trauma trial teams could consider the findings to develop targeted, behaviourally focused, solutions to the challenges identified in order to enhance the set-up and conduct of trials in this setting.

**Trial registration:**

NCT04145271. Trial registration date: October 30, 2019. Note that this paper does not report results from a specific trial but does include participants who were involved in the conduct of a registered pre-hospital feasibility study.

**Supplementary Information:**

The online version contains supplementary material available at 10.1186/s13063-023-07184-5.

## Introduction

Trials conducted in pre-hospital trauma are associated with a number of specific logistical and methodological challenges, which may have resulted in relatively few trials being conducted in this setting. For example, randomisation and intervention delivery are often difficult because they occur in remote locations, public spaces, private residences, or emergency vehicles (ambulance, helicopter and/or fast response car) [1]. Pre-hospital trauma trials in the UK may also face challenges in relation to the range of organisations, and their potentially competing priorities, when tasked with trial delivery (e.g. Air Ambulance charities that are separate from the National Health Service (NHS)). Methodological considerations related to the small number of potentially eligible patients available to recruit within pre-hospital trauma trials can also pose additional challenges related to design [2–4]. There are also ethical considerations associated with recruiting individuals who lack capacity to consent but require emergency pre-hospital treatment [5–8].

Previous studies have explored clinicians’ perspectives of the challenges and opportunities for the delivery of pre-hospital trials, but these have tended to focus on individual trials rather than the perspectives of clinicians involved in multiple trials across the pre-hospital trial landscape. A significant focus to date has also been on the experiences of emergency medical service personnel, largely paramedics, in land-based ambulance crews [1, 2, 8, 9]. Few studies have considered the challenges of conducting pre-hospital trauma trials that involve Helicopter Emergency Medical Service (HEMS) teams, yet many pre-hospital trauma trials are delivered by these services and involve doctors and paramedics delivering the trial intervention [4].

Pre-hospital trauma trials involve various people (e.g. doctors and paramedics) carrying out specific behaviours to accomplish tasks such as patient assessment, diagnosis, treatment priorities, intervention delivery and transportation to hospital. These behaviours are often carried out in difficult environments, can be compounded by poor weather conditions, and are distanced from in-hospital medical support [10]. Behavioural science offers a systematic approach to understand and change behaviours amongst healthcare practitioners [11]. More recently, the application of behavioural science to problems of the trial process and delivery has received attention [12–16].

The aim of this study was to use methods from behavioural science to identify the key challenges and opportunities involved in the set-up, design and conduct of trials in a pre-hospital trauma setting generally, and also to address the feasibility of a specific pre-hospital trauma trial. We aimed to understand the issues that affect the conduct of pre-hospital trials by garnering views from pre-hospital trial researchers involved in multiple trials across the UK and clinical investigators from a specific pre-hospital feasibility study.

## Methods

### Study design/participants

The study involved semi-structured interviews with two cohorts of participants: the first cohort were individuals who had been involved in pre-hospital trials (both trauma and non-trauma) in either a strategic or operational capacity (across different roles) within the UK — referred to herein as ‘Pre-hospital trial researchers’. This cohort was sampled to assess the broad challenges across pre-hospital trials. These interviews were intended to be open and without an *a priori* specific behaviour of interest and as such data collection was not structured around a theoretical framework. However, we did apply the Capability Opportunity Motivation-Behaviour (COM-B) framework to inform the data analysis (process outlined below). The COM-B framework was developed to understand the factors that influence behaviour, namely in relation to an individuals’ capabilities (physical/psychological), opportunities (physical/social environmental factors) and motivation (reflective/automatic) [11].

The second cohort were individuals involved in the recruitment and intervention delivery to the P-PRO study** (**Pre**-**hospital Zone 1 Partial Resuscitative Endovascular Balloon Occlusion of the Aorta for injured patients with exsanguinating sub-diaphragmatic haemorrhage) — referred to herein as ‘P-PRO clinical investigators’. P-PRO is an observational cohort study investigating the feasibility of pre-hospital Zone 1 partial Resuscitative Endovascular Balloon Occlusion of the Aorta (P-REBOA). REBOA is a complex intervention that involves the insertion of an aortic balloon occlusion catheter into the thoracoabdominal aorta. REBOA is not routinely available within the UK in either the pre-hospital or in-hospital setting. However, this intervention is currently being evaluated in the Emergency Department setting by the UK REBOA Trial. Pre-hospital REBOA has been in use by London HEMS since 2014 targeting the distal aorta (Zone 3), for the management of severe pelvic haemorrhage [12, 13]. Zone 1 P-REBOA describes an evolution of this technique involving balloon positioning in the thoracic aorta for patients with sub-diaphragmatic haemorrhage and severe, immediately life-threatening shock, combined with a procedural adaptation to allow some blood flow past the balloon.

In the P-PRO Study, London HEMS teams are tasked with deploying Zone 1 P-REBOA in patients in the pre-hospital setting. The balloon is positioned above the level of suspected haemorrhage and inflated to augment aortic blood pressure above the balloon whilst reducing bleeding below. Once the haemodynamics have stabilised, the balloon is partially deflated to allow some controlled distal blood flow (P-REBOA) and therefore oxygen delivery, to mitigate against the ischaemia that is otherwise created. P-PRO clinical investigators then transport patients to the nearest Major Trauma Centre, whilst managing the balloon in situ.

Interviews with the P-PRO clinical investigators were narrower in focus with specific behaviours of interest to be investigated (recruitment and intervention delivery in a future trial). As such data collection and analysis was informed by the Theoretical Domains Framework (TDF). The TDF is an established behavioural framework that integrates 33 theories of behaviour into 14 domains that inhibit or enable behaviour (knowledge, skills; social/professional role and identity; beliefs about capabilities; beliefs about consequences; optimism; reinforcement; intentions; goals; memory/attention/decision processes; environmental context and resources; social influences; emotion; behavioural regulation) [17]. Domains of the TDF can be mapped onto components of the COM-B framework [12].

### Behavioural specification: Action, Actor, Context, Time (AACTT)

For the interviews with P-PRO clinical investigators, we applied the Action, Actor, Context, Target, Time (AACTT) framework [18] to define the behaviour(s) of interest, inform sampling, and the design of the interview topic guide. Two individuals (1 paramedic and 1 consultant) contributed to a team discussion to gain appropriate contextual information pertaining to the P-PRO study and the AACTT components. See Additional file 1.

An AACTT analysis was not completed for the interviews with pre-hospital trial researchers because they were not conducted with a specific behaviour of interest in mind. As stated previously, they were more open to identify the scope of relevant behavioural influences across multiple pre-hospital trials.

### Data collection

Data collection was conducted from October 2021 to January 2021. Sampling was informed by the key principles of information power [19]. Email invitations were distributed to eligible individuals. A member of the research team (LL) then scheduled a mutually convenient time for a Microsoft Teams interview [20]. Interviews were guided by topic guides, informed by the TDF for the P-PRO clinical investigator interviews (see Additional files 2 and 3). We also presented P-PRO clinical investigators with a vignette that provided a clinical description of a potential REBOA-eligible patient in a pre-hospital context. We probed participants regarding their decisions to randomise the patient in this hypothetical scenario. A female researcher with a background in psychology led the interviews (LL), with KG (Trials Methodologist) present in half of the interviews conducted. LL and KG debriefed after each interview to review highlights relevant to the study, establishing key points to consider for analysis. Interviews were audio recorded and transcribed. Informed verbal consent was obtained from all participants. Participants were issued with an information sheet prior to providing verbal informed consent. Interviews were transcribed verbatim by an external transcription company. This study was approved by London—Southeast Research Ethics Committee (20/LO/0013).

### Data analysis

#### Interviews with pre-hospital trial researchers

A thematic data-driven inductive approach to analysis was applied [21]. One researcher (LL) re-read the interview transcripts and generated codes in NVivo [22] and Microsoft Excel [20] which described relevant features of the data prior to collating into themes. Themes summarised the content of interviewee responses and represented salient issues that were articulated by multiple participants. Following review/refinement of themes, a thematic framework was developed (LL, reviewed by KG) to facilitate data analysis. A double coder (KG) checked the themes and accurately described the content of participants’ responses in three interview transcripts. Coding discrepancies identified were discussed to reach consensus. Following the completion of the analysis, we mapped identified themes to the appropriate categories represented in the COM-B model and selected illustrative extracts for each theme.

#### Interviews with P-PRO clinical investigators

A TDF coding guide was used to aid data interpretation: this was developed and iteratively updated during the coding process (LL, ED (Health Psychologist), KG). One researcher (LL) coded transcribed data into the relevant TDF domains. Three interview transcripts were independently double coded. All coding discrepancies were resolved via discussion between coders (LL and KG). After coding data into TDF domains, belief statements (representative descriptions of utterances across participants) were generated (LL) [23]. Belief statements detailed how each domain may influence the behaviours of interest, namely (1) recruitment of patients to a future pre-hospital trial of REBOA and (2) delivery of the intervention. We refined belief statements further to specify how the barriers and enablers were thought to influence patient recruitment and intervention delivery in a future trial of pre-hospital REBOA.

Established TDF analysis methods were applied to identify the domains that were most likely to influence the target behaviours [23, 24]. We also developed overarching themes that described the content of related belief statements and domains to effectively summarise the findings. Overarching themes were initially generated by LL who assessed the belief statements across all domains and categorised them into separate themes. These themes and belief statements were refined by KG to ensure that they summarised the data accurately. Themes and TDF domains were then mapped onto components of the COM-B framework.

#### Patient and public involvement

A patient representative (VL) provided feedback on the design and findings of this study, as well as advice regarding dissemination.

## Results

### Sample characteristics

#### Pre-hospital trial researchers

A total of 7 participants were interviewed. All participants were/had been involved in pre-hospital trials in some capacity (e.g. strategic or operational design and delivery at local and/or national levels). Participants included 6 men and 1 woman and ages ranged from 39 to 53 (median = 49). Four participants disclosed their years of experience working within their roles (ranged from 2 to 13 years, median = 8).

#### P-PRO clinical investigators

A total of 16 P-PRO clinical investigators were interviewed, including paramedics (*n* = 5), emergency medicine consultants (*n* = 7), registrars (*n* = 2), a trauma surgeon (*n* = 1) and an anaesthetist (*n* = 1). This sample included 13 men and 2 women aged 34–50 (median = 42). Years of experience within their roles ranged from 1 to 20 years (median = 5).

### Findings

Findings from the pre-hospital trial researcher interviews are presented first below, prior to outlining the barriers and enablers identified from the P-PRO clinical investigator interviews.

#### Interviews with pre-hospital trial researchers: overarching challenges and opportunities across the field

We identified four core themes, which detailed the barriers and enablers of conducting pre-hospital trials across the UK — some findings were specific to trauma trials and others were more generic for pre-hospital trials. The four themes are presented in detail below (with associated COM-B component in parentheses) and included: Lack of an established research culture; environmental challenges and opportunities; infrastructure influences; and motivation to participate influenced by clinical equipoise.

##### Lack of an established research culture (COM-B: physical and social opportunity)

Some participants described a lack of research culture that constructively supported and developed staff to deliver research in the pre-hospital setting. Many participants indicated that pre-hospital staff, namely paramedics, were focused on service delivery as opposed to the additional work required to conduct trial-related research activities. Some referenced how the lack of dedicated research staff in the pre-hospital setting impacted the training required to deliver interventions in pre-hospital trials, as well as a lack of knowledge and understanding around the research process amongst pre-hospital staff.

...there’s so few of them [paramedics] that have actually been through the [research] process before…there isn’t that collective understanding that you rely on to a large degree within major specialties in the hospital, so that’s back to culture of not having that background in research as well. Pre-hospital trial researcher 1.

However, one participant indicated that the culture was changing, citing examples of an active pre-hospital trainee-operated research network (PHOTON) that provides opportunities for trainees to get involved in pre-hospital research. Another participant also cited positive experiences of conducting pre-hospital trials with paramedics, indicating that paramedics tended to be motivated to participate in clinical research for their professional development.

…paramedics were very keen to be involved in research because actually it was something that they could own, they could develop from their own professional perspective and also from their cohort… Pre-hospital trial researcher 2.

##### Environmental challenges and opportunities (COM-B: physical opportunity and physical/psychological capabilities)

In addition to the cultural challenges outlined, participants described other environmental barriers related to conducting pre-hospital trials. Specifically for trauma trials, this included comments related to the lack of eligible patients who require the clinical interventions (limited physical opportunity to recruit), which meant that it was also difficult for staff to retain the appropriate technical skill sets required to deliver trials (including intervention delivery, challenging physical capabilities). Working in a pressurised, fast-paced, often under-staffed, environment could challenge their mental ‘bandwidth’ impacting psychological capabilities to conduct pre-hospital research within this setting.

…the research procedures, themselves, may delay delivery of specific aspects of care, or may complicate the care to such an extent that those things that are being done are not done properly. It is a small team, potentially working in a difficult environment with very little bandwidth. Pre-hospital trial researcher 7.

Linked to this bandwidth issue, the requirement to design pre-hospital trials with minimal paperwork to reduce the associated workload pressures was also emphasised.

... it’s a pressured environment and if we want to get trials off the ground, deliver trials and recruiting in an efficient way, we need to keep the [administrative] demands to a minimum. We’re meant to make it easy for our staff to recruit patients. Pre-hospital trial researcher 6.

The logistics around intervention allocation in a pre-hospital environment was also noted as a barrier: many emphasised the difficulties associated with concealing allocation outcome and access to online platforms designed to facilitate randomisation in remote areas. Consideration of the pre-hospital trauma environmental context in relation to consent (i.e. research without prior consent) and how this intersects with cultural or religious considerations and the pace of decision-making required was also identified as a notable challenge to trial conduct.

…we spent a fair amount of time in various motorway service stations up and down the country chatting to Jehovah’s Witness hospital liaison people about how not to enrol a Jehovah’s Witness without their consent. Pre-hospital trial researcher 2.

Other operational challenges linked to follow-up in relation to patients being randomised to a trial within a pre-hospital setting, but transported to a receiving hospital that was not set up to deliver the trial.

…there were occasions when patients were deemed so critically ill that they take them to the nearest hospital. If those nearest hospitals weren’t part of your study, getting as we would be doing professional legal or personal legal representative consent became difficult or impossible, and so we lost a number of patients to follow up because we had no way of obtaining consent. Pre-hospital trial researcher 4

…because I guarantee the first patient you recruit will go [sic] another trauma centre and then you’ll have randomised the patient but have no means to capture the follow up data. Pre-hospital trial researcher 3

Nevertheless, participants also indicated the opportunities of conducting trials in a pre-hospital setting — the ability to deliver time-sensitive interventions to patients before they arrive in the hospital was regarded as an important implication of pre-hospital care.

A lot of interventional treatments in a variety of different conditions are time-sensitive, say heart attacks, there’s a lot of evidence out there that treatments are time-sensitive. The earlier you get them, the better the benefit…so there’s definite benefit in moving treatment forward. That’s the big advantage of pre-hospital trials, is that they are working in that environment before they arrive in hospital… Pre-hospital trial researcher 5.

##### Infrastructure influences (COM-B: physical opportunity, social opportunity, automatic motivation)

As well as the broad environmental challenges and opportunities outlined, participants also referred to organisational practices that impacted on opportunities to accrue patients. Differences between organisations and institutions could create difficulties during the conduct of pre-hospital trials, namely in relation to the allocation of recruitment accruals, and associated financial reward provided through clinical research networks. For example, patients may have been recruited through an ambulance service but the accrual recognition (and any associated resource reward) would be awarded to the secondary care hospital which they were transported to.

… some of the big pre-hospital trials, all of the credit went to the hospitals that took patients afterwards, even though all the work was done in pre-hospital care, and that’s still an ongoing problem… Pre-hospital trial researcher 1.

Furthermore, the fact that Air Ambulances are independent charities rather than NHS organisations was also described as creating barriers to the set-up and conduct of pre-hospital trials. Air ambulances were described as having different priorities towards pre-hospital research, motivated largely by future funding to deliver innovative care.

… if you’re considering any trial involving air ambulances, you have to be so, so cognisant of in all of your negotiations that they are not NHS organisations, they are … independent charities whose sole job is to raise money to allow the pre-hospital teams to provide an enhanced level of care … Pre-hospital trial researcher 2.

##### Motivation to participate influenced by clinical equipoise (COM-B: reflective motivation and social opportunity)

Motivations of clinical staff to participate in pre-hospital trials were often referenced in relation to clinical equipoise. A lack of clinical equipoise was referenced as particularly problematic during the conduct of pre-hospital trials due to institutional biases that motivated the delivery of interventions viewed as ‘innovative’ by external organisations. Beliefs about a trial intervention amongst clinical teams could also change overtime, which was sometimes reported to be linked to the influence of other people or organisations.

Then the other very significant thing was because it took us a long time to get that first cluster open if you like, during that time people started to lose equipoise and started to read more into the military data on administering [intervention]. The important thing to remember as well is that a lot of the clinicians that staff pre-hospital services are in the military. So not only have they seen it first-hand they also… they’ve bought into it. Pre-hospital trial researcher 3.

Following on from these perspectives and considerations about pre-hospital trials in general (including trauma), we next highlight the findings from the interviews with P-PRO clinical investigators. These interviews were designed to examine the feasibility of conducting a trial that examines the effectiveness of REBOA in a pre-hospital setting.

#### Interviews with P-PRO clinical investigators: specific challenges and opportunities for a pre-hospital trial of REBOA

The following section describes the themes, and associated behavioural influences, that were identified as relevant for the delivery of pre-hospital REBOA within a Randomised Controlled Trial (RCT) and the feasibility of conducting a trial in this setting. A total of 7 themes were identified: Views about pre-hospital REBOA: Individual and Collective Equipoise; Determining patient eligibility in a rapidly changing clinical setting; Technical and non-technical skills required to recruit patients and deliver REBOA; Staff availability and team dynamics influence recruitment and intervention delivery; Targets related to future trial recruitment; Emotional influences on recruitment and intervention delivery; and Challenges of the pre-hospital environment. Each of these themes is presented in detail below with relevant TDF domains in parentheses. An extended table containing the frequency of the TDF domains and belief statements is provided in Additional file 4.

##### Views about pre-hospital REBOA: individual and collective equipoise (TDF beliefs about consequences; social influences)

Participants recognised the potential benefits associated with REBOA in the pre-hospital setting, but most noted the potential risks involved. There were mixed responses in relation to questions that explored equipoise and relatedly whether clinical participants would be happy to randomise patients to a pre-hospital trial of REBOA. Whilst some participants indicated that they would hesitate to randomise, many suggested that their inherent positive beliefs about REBOA (and lack of equipoise) meant that they would struggle to follow the randomisation outcome if the patient was allocated to standard care.

Whilst I’ve said ultimately we don’t know whether our patient would have survived without us I think we’re going to have a group of patients who are literally therefore dying in front of us and maybe if we were in an RCT we wouldn’t have put a balloon in or we wouldn’t have inflated a balloon. Once you’ve gone down this road you could end up feeling that you’ve denied a patient some care that could have saved them. Consultant, participant 4.

Relatedly, many participants highlighted the overall community equipoise, often noting the willingness to participate in a trial of pre-hospital REBOA to establish the effectiveness, which could influence personal and team decisions.

There’s passionate people who are very pro and people who aren’t, but I suppose the thing is that the vast majority of clinicians who are involved in this also understand the importance to the population of these patients to answer this question. Consultant, participant 8.

##### Determining patient eligibility in a rapidly changing clinical setting (TDF beliefs about consequences)

Linked to the hesitancy in delivering the REBOA intervention in a future trial as emphasised above, the challenges associated with determining patient eligibility in relation to the rapidly changing clinical setting was also noted. For example, the clinical picture of the patient in a pre-hospital setting often changes quickly, which means that patient eligibility to participate in a trial could be fluid and changeable. Participants reported that assessing eligibility required a level of flexibility and attentiveness to the status of the patient. This was particularly notable in the pre-hospital environment, which was described as dynamic and unpredictable, a factor which could also affect clinician confidence to determine patient eligibility and perform REBOA out with the hospital in a future trial.

It’s very fluid, it’s very dynamic, there’ll be certain situations where you start down a trajectory or a path of treatment and actually when you’ve reassessed, not that it’s incorrect, but actually that path slightly changes and they might respond well to something that then stops you on that path or just slows you down that path… Paramedic, participant 3.

##### Technical and non-technical skills required to recruit patients and deliver pre-hospital REBOA (TDF skills; memory, attention and decision-making)

Participants highlighted the delivery of REBOA to be a technical skill which could often be difficult to maintain (in a trial), given the infrequency of eligible cases. As well as the technical, specialist skills required to conduct REBOA, participants also highlighted the non-technical skills required to effectively recruit patients and deploy the intervention in a pre-hospital setting. For example, communication skills were noted as essential, particularly in fast-paced pre-hospital environments whereby potential distractions could challenge the provision of patient care. The ability to make fast-paced decisions, such as the decision to randomise a patient, was also emphasised given the dynamic nature of the environment.

…But the things that we do to try and bring any scene under control are the same that we do whether it’s a P-PRO or not, which are all about communication, primacy of care, dissemination of plan, role allocation, you know? Scene safety… it all comes down to… the unique skill set of pre-hospital care is just communication, you know? There’s no technical skill. And the ability to have been in enough scenes that you’ve got bandwidth to deploy that communication, and actually that’s not unique to REBOA or the P-PRO scenes. Consultant, participant 7.

##### Staff availability and team dynamics influence recruitment and intervention delivery (TDF environmental context and resources; social influences)

Participants described the challenges of working in a pre-hospital setting with reference to the lack of available staff with the required technical skills to support trial recruitment and intervention delivery during some shifts. Staff rotation schedules also meant that staff would be unlikely to work with the same team during each shift (also raising issues related to trial training) and team dynamics impacted on study delivery in terms of communication and managing roles. Some participants emphasised that trials evaluating pre-hospital REBOA may require additional personnel to facilitate randomisation. A lack of staff available to support pre-hospital activities and recruitment could potentially limit the conduct of a trial in this setting.

… I think three or four [staff members] is a really nice number because there’s quite a lot going on, and I suppose some of it may be equally the HEMS team delivering the procedure … what’s the number to facilitate the randomisation of the patient group, is it two or is it three, because at some point to randomise you’re going to have to… facilitate the randomisation. Paramedic, participant 5.

##### Targets related to future trial recruitment (TDF goals)

Whilst goals and targets for recruitment in the context of a trial was often deemed necessary, many emphasised how it may negatively impact staff who were already operating in a stressful pre-hospital environment.

…But yeah, it needs to be left to the investigators, but for people on the day to day, I think the pressure of recruiting a number shouldn’t have any influence on the decision-making process and there’s a problem if it does. I’m not sure entirely how helpful they [recruitment targets] are. Consultant, participant 7.

##### Emotional influences on recruitment and intervention delivery (TDF emotion)

In relation to the fact that pre-hospital environments were often described as stressful, many participants indicated that they were worried about conducting the REBOA procedure and managing the (often chaotic) situation appropriately, particularly generating the correct decisions in uncertain and unpredictable environments. When probed about randomising a patient to a future pre-hospital trial of REBOA, many participants indicated that the emotional burden related to decision-making and the conduct of the intervention would remain:

…It’s difficult to take away that human side when you… and certainly some of these patients have a high conscious level initially and they just… the conscious level just gets worse and worse and you’ve kind of made that emotional contact. Paramedic, participant 5.

##### Challenges of the pre-hospital environment (TDF environmental context and resources)

All of the barriers outlined above were compounded by the challenges associated with operating in a pre-hospital environment. This included difficulties associated with deploying REBOA when the weather was poor, the lighting was dim and there were multiple people present (e.g. such as police and other members of the public). These barriers could deter effective randomisation or the delivery of the intervention in a future trial. Furthermore, their distance from the hospital often influenced decisions to recruit. The distance from a hospital was predicted to determine decisions to randomise patients, namely due to the trajectory of the patient’s status.

…we go on a helicopter and we sometimes get to people in ten, 15, 20 minutes and they’re almost dead, it’s harder to look at that person and say… if it tells me not to put a balloon in I’m happy with the next 40 minutes, or even if I’m unbelievably quick and it’s 20 minutes, am I really happy with that without intervening? That doesn’t necessarily mean we shouldn’t do an RCT, but I think we might find that the alternative is quite invasive in those patients, i.e. maybe a thoracotomy or something like that. But the time does have an effect because when we get there quicker and they’re that sick, that’s a very different patient to an hour down the line and they’re that sick. Consultant, participant 6.

##### Comparison of findings across pre-hospital trial researchers and P-PRO clinical investigators

**Fig. 1:**
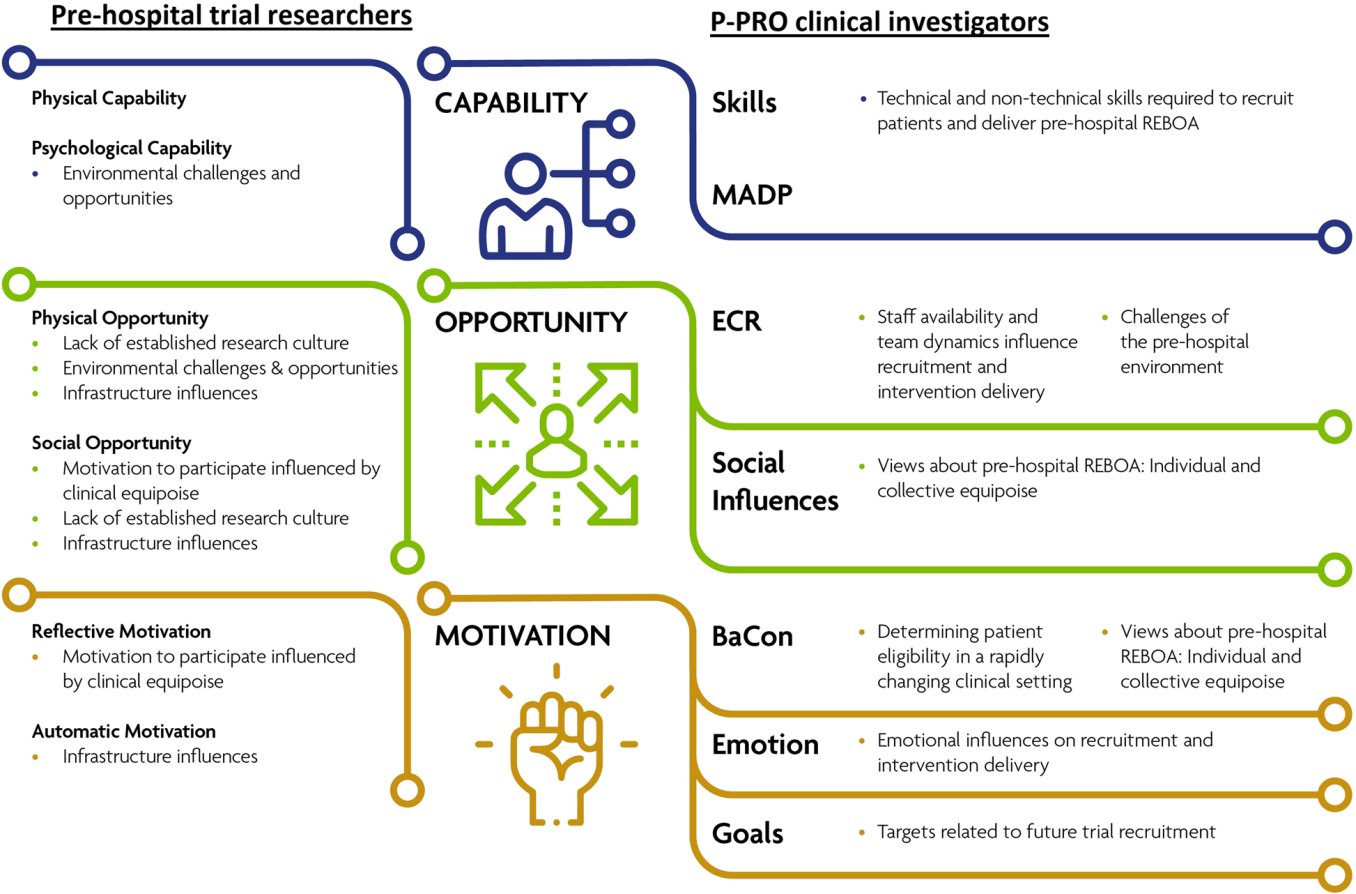
Themes linked to the COM-B and TDF frameworks. *Note*. The themes presented on the left represent themes reported by the pre-hospital trial researchers, whereas the themes depicted on the right indicate topics that arose from interviews with P-PRO clinical investigators. MADP, memory, attention and decision-making processes; ECR, environmental context and resources; BaCon, beliefs about consequences

Comparative analysis of the themes identified across both cohorts of participants revealed several areas of overlap between the data sets. Figure 1 depicts the linkage of themes identified from the COM-B analysis of the pre-hospital trial researchers linked to the TDF domains identified in the P-PRO clinical investigator interviews. Whilst some themes were represented by more than one COM-B component, most of the themes were represented by the COM-B component ‘Opportunity’. Across both groups of participants, physical opportunity challenges related to the environmental context of conducting pre-hospital trials. Namely, the research backgrounds of pre-hospital staff, team dynamics within a rotating schedule, involvement of external organisations, and the infrequency of eligible cases could affect the trial design, set-up, and conduct.

## Discussion

This study has highlighted a number of challenges and some opportunities for consideration by trial teams conducting pre-hospital trauma trials. These challenges and opportunities exist across health care systems and include individual (e.g. motivation/beliefs), organisational (e.g. research culture) and broader societal (e.g. legal/legislative) considerations. There were considerable areas of overlap between the pre-hospital trial researchers (who identified overarching challenges across the field) and the P-PRO clinical investigators (who provided more detailed challenges for a case study pre-hospital trauma trial), providing further validation across the data sets. The approach we used provides a behavioural diagnosis of the key challenges across the system that impact on the successful delivery of pre-hospital trauma trials.

For the pre-hospital trial researchers, the lack of an established research culture within the pre-hospital environment was identified as a key challenge to the conduct of pre-hospital research. Research activity was regarded as secondary to immediate patient care, and often difficult to factor in due to operational pressures. Indeed, paramedics have reported that their involvement in pre-hospital research is not featured as part of their professional responsibilities, and that time pressures/constraints often limit their participation [25]. In other clinical specialties, various initiatives have been developed that are designed to enhance research participation amongst clinicians and medical students [26]. However, the situation in pre-hospital research is changing with the establishment of PHOTON, which includes a range of trainee clinical roles, and who have a remit to improve the quality of pre-hospital research [27].

The additional ‘work’ of trials was also identified in both of our interview cohorts in relation to trial conduct. The importance of designing pre-hospital trials with minimal paperwork to reduce the participation burden for pre-hospital staff was identified in our study, both directly and indirectly (i.e. limited bandwidth) and has been highlighted in other pre-hospital trials, as well as the logistical challenges related to handover procedures with regards to data collection [1]. Lazarus et al. [10] reported that patient enrolment to the PARAMEDIC-2 trial was often constrained by contrasting views and opinions of paramedics who were not involved in the trial. Linked to this, participants in our study reported that some pre-hospital staff may be more focused on service delivery as opposed to the additional work that trials present. Having the psychological capacity, or bandwidth, to take on the additional work of trials in a pre-hospital trauma setting was also raised as important. Whilst some of these challenges are clearly context-specific, the overall concept of the additional ‘work’ of trials has been cited in other studies [28].

We used behavioural frameworks (COM-B and TDF) to facilitate our understanding of the barriers and enablers to conducting pre-hospital trials. Although this approach is gaining traction in the field of trial methodology [15, 16, 29–35], few studies have applied behavioural approaches to understand trial challenges within a pre-hospital context. In a clinical care pre-hospital setting, an interview study used the TDF to explore the factors that influence paramedics’ administration of tranexamic acid [36]. Multiple influential factors were identified, which provided an avenue for the development of interventions designed to address challenges and maximise the enablers identified. This is a key advantage associated with the use of behavioural frameworks such as the COM-B and TDF. Barriers/enablers that are rooted in key domains (e.g. TDF Social Influences) or components (e.g. COM-B motivation) can be mapped onto evidence-based interventions through established methods in the field of behavioural science [12]. The current study demonstrates how behavioural approaches can also be used to address questions related to trial feasibility in a pre-hospital setting, both across the specialty and in a specific case study. Specific solutions to address feasibility challenges can be developed on the basis of these findings, developing bespoke strategies to ensure the success of a future pre-hospital trial [37].

### Strengths/limitations

Our findings are based on reports from research staff with experience of working within a range of pre-hospital trials, as well as staff occupying diverse roles (e.g. paramedics, consultants, registrars) within a pre-hospital trauma observational cohort study (the P-PRO study). Our findings therefore provide insight into the factors that affect trial processes using examples from multiple trials in the pre-hospital setting. Nevertheless, our sample comprised of individuals who were largely supportive about a future trial examining pre-hospital REBOA, particularly the P-PRO clinical investigators who were generally enthusiastic about REBOA. There may have been other factors identified if we had interviewed individuals who were more sceptical about REBOA or pre-hospital trauma trials more generally. Furthermore, the use of two different topic guides for our two cohorts of participants could be regarded as a study limitation. We did not collect any data pertaining to the clinicians’ experience in recruiting participants and how this affected their confidence in recruiting to trials (particularly in emergency, pre-hospital and critical care research). This might be important to explore in future research.

## Conclusion

This study has highlighted that pre-hospital trauma trials face many context-specific but also generic challenges to their successful delivery. These challenges were identified across multiple levels, within systems, organisations, and individuals. Future pre-hospital trauma trials could consider the findings to develop targeted solutions to the challenges identified in order to enhance the design, set-up and conduct of trials in this setting.

## Supplementary Information

**Additional file 1.** Table that details the AACTT components.

**Additional file 2.** Interview topic guide for the pre-hospital research staff.

**Additional file 3.** Interview topic guide for the P-PRO clinical investigators.

**Additional file 4.** Table of TDF domains and belief statements. Provides a summary of the content within all TDF domains, with example quotes and associated frequency data.

## Data Availability

No data available for sharing beyond those published.

## Abbreviations

RCT: Randomised controlled trial
HEMS: Helicopter Emergency Medicine Service
PHEM: Pre-hospital Helicopter Emergency Medicine
NHS: National Health Service
REBOA: Resuscitative Endovascular Balloon Occlusion of the Aorta
P-PRO: Pre-Hospital Zone 1 Partial Resuscitative Endovascular Balloon Occlusion of the Aorta for injured patients with exsanguinating sub-diaphragmatic haemorrhage
TDF: Theoretical Domains Framework
COM-B: Capability, Opportunity, Motivation – Behaviour
AACTT: Actor, Action, Context, Target, Time
CTIMP: Clinical Trials of Interventional Medicinal Products
PHOTON: Pre-hospital Trainee Operated Research Network
