# Supplementary material for: Challenges and opportunities for conducting pre-hospital trauma trials: a behavioural investigation
Source: Trials. 2023 Mar 2;24:157. doi: 10.1186/s13063-023-07184-5 (PMC9983243; doi:10.1186/s13063-023-07184-5)
Supplement: Supplementary file 1 — Additional file 1. Table that details the AACTT components. [file 13063_2023_7184_MOESM1_ESM.docx]

The AACTT components of the PPRO study.

| AACTT Component | PPRO-Behave study |
| --- | --- |
| Action(s) | *Recruitment* – standard clinical assessment of the patient to determine eligibility. Includes establishing mechanisms of injury and liaising with the team. The decision to recruit a patient typically lies with the consultant.  *Intervention delivery -* insertion of a REBOA catheter through the femoral artery (cannulation). |
| Actor(s) | The HEMS team – 2/3 people (usually 1 or 2 doctors and a paramedic, sometimes a registrar). |
| Context | *Physical setting* – outdoors, can be in turbulent conditions, in a public place (e.g. train station) or indoors in a private residence.  *Emotional setting* – stressful, unpredictable, can be situated in a violent scenario (e.g. cases that involve violence).  *Geography* – distance from hospital can vary. |
| Target | Patients with rapid life threatening bleeding (eligible for the REBOA intervention). |
| Time | Any time of day. Most commonly after school, and around 11pm-1am on Friday/Saturday nights. |
