# Supplementary material for: Challenges and opportunities for conducting pre-hospital trauma trials: a behavioural investigation
Source: Trials. 2023 Mar 2;24:157. doi: 10.1186/s13063-023-07184-5 (PMC9983243; doi:10.1186/s13063-023-07184-5)
Supplement: Supplementary file 2 — Additional file 2. Interview topic guide for the pre-hospital research staff. [file 13063_2023_7184_MOESM2_ESM.docx]

**This topic guide will be used in a flexible manner to generate discussion between prehospital trial research teams and the researcher. This topic guide may be subject to refinement in response to interview findings.**

***Instructions for interviewers:***

Inform participants of your name and role at the University of Aberdeen. Go through the main points within the Participant Information Leaflet. Provide the opportunity to ask questions. Clarify about anonymisation procedures. Highlight that the session will be recorded, and the audio recording will only be used for transcription and data analysis (after gaining permission, start the recorder). Go through the consent form and ask participant for their consent.

**Brief intro to purpose of study and progress so far.**

| 1. What is your gender? You can prefer not to say. |
| --- |
| 1. What is your job title? |
| 1. How long have you been a (job title)? Years. How long have you been involved in trials more generally? |
| 1. What is your age? Are you happy to tell us your age |

**Questions for prehospital trial researchers (questions will be adaptable and flexible based on individual staff roles etc)**

- **Tell me about your experience of working in clinical trials? Prompt: how did that lead you to the role that you now have? Have you ever been involved in prehosp trials?**
- **Tasks/activities as part of that role**
  - In your role, what is your perspective about the **challenges** for delivering **trials**? Prompt: How does that change for **prehospital trauma trials**. Recruitment? Patient identification? Randomising out in the field?
  - What are the **opportunities** to delivering prehospital trials?

What are the things you think could **enhance the opportunities/mitigate the challenges** to delivering **prehospital trials**? Solutions?

- **How easy/difficult is it to run these trials across the UK?**
- How easy/difficult was it for trial sites to successfully identify and then recruit participants?
- Do you think there is anything that encourages or discourages sites to consider potential participants for [specific trial]? And what about prehospital trials more generally?
- What were the generic problems encountered when recruiting participants? And what about specific problems related to recruitment?
  - Did the trial adopt any strategies to help address this?
- **Any exemplars that work particularly well?** Are you familiar with the UK REBOA trials currently running from Aberdeen? If no – explain – if yes – link to current study (PPRO) – what do you think main challenges/opportunities would be?
- **What are the key things that you think sites would need to consider for a future large-scale trial of pre-hospital REBOA (pre-hospital trauma trial)?**
- What would the main challenges be?
  - What would the main opportunities be?

**Do you know of any other prehospital trials we could be investigating?**

**That’s all the questions that I have for you, is there something else you would like to say or expand on?** [Explore any other issues of relevance to participant not covered by the above before wrapping up discussion].

**Thank you very much for your time.**
