# Supplementary material for: Challenges and opportunities for conducting pre-hospital trauma trials: a behavioural investigation
Source: Trials. 2023 Mar 2;24:157. doi: 10.1186/s13063-023-07184-5 (PMC9983243; doi:10.1186/s13063-023-07184-5)
Supplement: Supplementary file 3 — Additional file 3. Interview topic guide for the P-PRO clinical investigators. [file 13063_2023_7184_MOESM3_ESM.docx]

**This topic guide will be used in a flexible manner to generate discussion between trial staff interviewees and the researcher. This topic guide may be subject to refinement in response to interview findings.**

***Instructions for interviewers:***

| 1. Do you identify as male or female? |
| --- |
| 1. What trial site do you work from? |
| 1. What is your job title? |
| 1. How long have you been a (job title)? Years. |
| 1. What is your age? |

Inform participants of your name and role at the University of Aberdeen. Go through the main points within the Participant Information Leaflet. Provide the opportunity to ask questions. Clarify about anonymisation procedures. Highlight that the session will be recorded, and the audio recording will only be used for transcription and data analysis (after gaining permission, start the recorder). Go through the consent form and ask participant for their consent. Highlight right to withdraw at any time. Emphasise that there are no right/wrong answers, we are interested in their experience.

| Questions for clinical staff involved in PPRO (questions will be adaptable and flexible based on individual staff roles etc) |
| --- |
| 1. Tell me about your role (in relation to PPRO)? Have you delivered REBOA in a prehospital setting? |
| 1. What are the tasks/activities you complete on a day-to-day basis? In relation to the PPRO study |
| 1. What are the steps involved in recruitment (for PPRO study? What about follow-up care? |
| 1. How do you and/or colleague(s) decide which patients will receive the intervention and be recruited to PPRO? |
| 1. Are there any skills required to recruit participants? What about for the delivery of pre-hospital REBOA? |
| 1. Have you received any formal training for recruitment and intervention delivery? |
| 1. How confident are you in recruiting participants for PPRO and/or (assisting in) delivering the intervention? |
| 1. Do you use any specific protocols, policies, or guidelines to guide your discussions for trial recruitment and intervention delivery? |
| 1. How easy/difficult is it to successfully identify and then recruit participants and deploy the intervention? |
| 1. Are there any problems that you usually encounter when you recruit participants and deliver the intervention? |
| 1. Can you give me an idea of the kinds of situations where it becomes difficult to consider potential participants? |
| 1. What would help you overcome these problems/difficulties? |
| 1. Can you describe how you feel when you recruit patients to PPRO? (e.g. guilt, fear, worry, concern or satisfaction)? What about when you are performing the intervention [if applicable to role]? Can you describe the emotional context? To what extent does this affect your decision to recruit and deploy REBOA? |
| 1. Are there specific aspects of your work environment that make it easier or more difficult for you to recruit patients for [PPRO] and deploy the intervention? 2. Are there any challenges/advantages to deploying the intervention/recruiting participants in a prehospital environment specifically? |
| 1. Do you have the resources available to help with delivering the intervention and recruiting participants? |
| 1. How do the views of others influence whether a participant is recruited and the intervention is deployed? |
| 1. How would you describe your personal motivation for recruiting participants to [PPRO] and delivering the intervention (or assisting in the delivery)? |
| 1. Is there anything that encourages you or discourages you to consider potential participants for the [trial/study]? What about for delivering the intervention? |
| 1. Are there any (personal) strategies that you use to assist in the recruitment and intervention delivery process? |
| 1. Are there any specific targets/quotas that you need to meet for recruitment? |
| 1. Do you receive any feedback about the ways in which the intervention has been deployed (or how you have assisted with the delivery of it)? What about feedback for recruitment generally? |

**Now I would like to ask more broad questions regarding a future randomised trial of PPRO. It can be helpful to also consider what we have already covered in your responses in relation to your experience of PPRO or other prehospital trials you have been involved in.**

| 1. Overall, do you expect eligible participants to be recruited to the study and receive the intervention? |
| --- |
| 1. What do you think are the overall benefits and downsides of recruiting participants to prehospital trials? What about for delivering the intervention? |
| 1. What do you think would happen if you were unable to successfully recruit participants to the trial and deliver the intervention? |
| 1. How important or unimportant do you feel it is to offer a pre-hospital REBOA service? |
| 1. What are the key things that you think sites would need to consider for a future large-scale trial of pre-hospital REBOA (pre-hospital trauma trial)?  - What would the main challenges be? Do you think any electronic devices would help to facilitate recruitment? Why/how? Would there be any downsides to this? - What would the main opportunities be? |

**That’s all the questions that I have for you, is there something else you would like to say or expand on?** [Explore any other issues of relevance to participant not covered by the above before wrapping up discussion].

**Thank you very much for your time.**

**Clinical Vignette**

***Participants will be presented with this vignette at the end of the interview and asked about what is key for considering this patients inclusion in the PPRO Study.***

You are called to a 19-year-old male patient who has been stabbed in the abdomen and groin. You arrive on-scene 14 minutes after the 999 call by fast response car. The LAS paramedic crew describe finding the patient lying in an alley way in a pool of blood, appearing agitated, with an armed response officer pressing on his groin and a CAT tourniquet applied to the L proximal thigh. He was witnessed to run to this location and collapse. On your arrival the patient is in the back of an LAS ambulance.

On examination you find two 3cm incisional wounds in the LUQ, one 3cm L sided periumbilical wound and one 3cm incisional wound on the L medial thigh 8cm below the Inguinal Ligament. The patient appears extremely pale, has an intermittently palpable, weak, low volume central pulse, HR 130bpm, an 18G cannula in his right ACF, unrecordable NIBP. His airway is patent, he has deep sighing respiration, bilateral air entry, and appears unconscious. On painful stimulation he localises and groans. POCUS examination reveals no pericardial blood and no haemothorax, FAST scan is positive. The transfer time to the Royal London Hospital is approximately 15 minutes.
