# Supplementary material for: Challenges and opportunities for conducting pre-hospital trauma trials: a behavioural investigation
Source: Trials. 2023 Mar 2;24:157. doi: 10.1186/s13063-023-07184-5 (PMC9983243; doi:10.1186/s13063-023-07184-5)
Supplement: Supplementary file 4 — Additional file 4. Table of TDF domains and belief statements. Provides a summary of the content within all TDF domains, with example quotes and associated frequency data. [file 13063_2023_7184_MOESM4_ESM.docx]

| **Domain (descriptions) of the TDF**  [Cane et al. Implementation Science 2012, 7:37] | **Belief statements** | **Frequency**  **(Utterances/**  **participants)** | **Sample quote (with participant number)** |
| --- | --- | --- | --- |
| **1**  **Environmental context and resources**  Factors related to the prehospital setting/environment/  person-environmental interactions that influence PPRO recruitment and intervention delivery.  Domain frequency: 14/14 | Staff availability and team dynamics influence recruitment and intervention delivery^[[1]](#footnote-1)^^[[2]](#footnote-2)^ | 14/7 | “I would certainly be more stressed trying to get the team to function if they were inexperienced than if you had a team that was kind of on it and you didn’t have to ask them to do things, or you didn’t have to show them how to do things.” S03L0092, Paramedic.  “In the pre-hospital scenario you haven’t got the same thing as in hospital where you have always got a trauma team and a number of people to do things truly at the same time.” S03L0095, Consultant. |
|  | Scarcity of REBOA cases influences recruitment and intervention delivery^12^ | 11/6 | “I guess for me, I find that there are not huge challenges in recruiting the patients, I find I just don’t happen to get the patients I can recruit.” S03L0101, Consultant.  “Again, speaking from the paramedic cohort, these kind of extreme patients, major trauma patients, in an average year we would see maybe one or two as a regular paramedic, maybe even one a year. This kind of 1% of major trauma patients.” S03L0093, Paramedic. |
|  | Distance from hospital influences recruitment and intervention delivery^12^ | 14/5 | “The things that affect your decision [to deliver REBOA] are the time to hospital.” S03L0095, Consultant.  “You’ve got to convince people, or remind people how long it actually takes to run to hospital because it does actually take some time to package and go and all of that and there’s a little bit of a crystal ball element I think, which is you don’t really know if they’re going to get worse or not until it happens, there’s all of that.” S03L0098, Consultant. |
|  | Environmental challenges such as weather and time of day influences recruitment and intervention delivery^2^ | 16/5 | “But we are often (inaudible - audio interference) patients with it raining, windy, we’re trying to put a drape over a patient and the wind just picks it up and moves all the equipment.” S03L0096, Paramedic.  “The environment is more, usually more difficult, not always but there’s often wind and rain and darkness and it’s an unpredictable scene and environment and even being really cold can make your fingers stiff and it’s harder to do it a fiddly procedure. So the environmental factors can’t be underestimated and it does make it quite challenging.” S03L0098, Consultant. |
|  | Training resources can impact recruitment and intervention delivery^12^ | 7/4 | “I don’t think there’s a problem in terms of resources. There’s good training, good training equipment, we’re well-equipped to deliver the intervention, even in a two-person team if needs be. I don’t feel there’s any limitation in terms of resource and training.” S03L0101, Consultant.  “The biggest challenge to education and training is the tail-end of it, what you do surrounding the deflation of the balloon and the handover, and then looking at making sure that we have got that feedback for outcomes at the end of it. I don’t think we will move forward with this unless individuals actually have an idea of what it looks like further on down the line.” S03L0103, Consultant. |
|  | Feedback processes influence recruitment and intervention delivery^12^ | 6/3 | “But the REBOA specific debriefs are very useful to see the physiology in the cold light of day and talk about the impact of physiology.” S03L0101, Consultant.  “I think… specifically with the PPRO REBOA candidate that we recruited, we had a very, very focussed debrief with PPRO leads, REBOA clinical leads, over and above what we would normally do with the governance process. It was an hour of really in-depth questioning about the decision, about how the patient was presenting in the patient trajectory, and that was really helpful to… it was almost immediately after, so it was like the next day, so the information was fresh.” S03L0093, Paramedic. |
| **2**  **Beliefs about consequences**  Perceptions about outcomes, and advantages and disadvantages of using REBOA in a prehospital setting.  Domain frequency: 14/14 | REBOA is (not) beneficial^2^ | 23/7 | “This intervention is not an end-point in their treatment, it’s not going to make anything better, but it’s going to buy them time for them to get to an operating theatre normally for their life to try and be saved.” S03L0091, Registrar.  “Then they do turn the corner and they do stabilise out of it, so it’s subjecting someone to REBOA, which has got significant risks, that’s a challenging decision.” S03L0103, Consultant. |
|  | Equipoise influences recruitment^12^ | 12/7 | “That’s the main challenge that their one of the higher acuity groups of patients that we see and we know that there is a route to hospital for them and we can’t actually hand on heart say we definitively know what’s going to happen if we don’t do the procedure on scene and therefore I think there’s a bit of bias potentially if you are like a real believer that the patient will never make it to hospital versus if you are not quite sure. I think there’s a risk that you’re biased by your involvement and your knowledge of it.” S03L0095, Consultant.  “You know, I’m reasonably positive about the procedure and therefore probably have inherent bias, and therefore if it was an RCT and I had someone young bleeding out and they were randomised away from REBOA, I would struggle with that.” S03L0101, Consultant. |
|  | Clinical picture of the patient can change^12^ | 2/2 | “Because the risks and benefits are incredibly tricky to really put your finger on, you know, there are trauma patients who respond well to blood and who look terrible to begin with, who you think would be a REBOA candidate. Then they do turn the corner and they do stabilise out of it, so it’s subjecting someone to REBOA, which has got significant risks, that’s a challenging decision.” S03L0103, Consultant.  “It’s very fluid, it’s very dynamic, there’ll be certain situations where you start down a trajectory or a path of treatment and actually when you’ve reassessed, not that it’s incorrect, but actually that path slightly changes and they might respond well to something that then stops you on that path or just slows you down that path…” S03L0093, Paramedic. |
| **3**  **Memory attention and decision process**  The processes involved, and the factors (e.g. a patient’s circumstances) taken into account, when they make the decision to recruit to PPRO and deploy REBOA.  Domain frequency: 14/14 | Determining mechanism of injury is a key step in the decision-making process^2^ | 21/9 | “Once you’ve decided what the injuries are the patient’s dying from it will be relatively obvious to the team about what zone therefore the balloon needs to go into.” S03L0091, Registrar.  “So the key elements are those collections of… it’s not just one thing, it’s the collection of evidence of severe exsanguination, there’s evidence of exsanguination there. That’s the key element for me.” S03L0103, Consultant. |
|  | Chaotic scenes often challenge our bandwidth^12^ | 6/5 | “One, that’s stressful, having to memorise something that you’re not using frequently and then it sits in the back, bugging you that maybe you’ve forgotten something important, so you have the confidence that someone else has got all that information if you need it.” S03L0111, Surgeon.  “I think the specificity of REBOA and PPRO is the bandwidth required to make the decision to deploy and then monitor, it’s deployment and the effect of its deployment. Effectively, you need someone to be pretty task-fixated on the REBOA.” S03L0101, Consultant. |
|  | PPRO requires fast paced decision making  (combine with skills)^12^ | 9/5 | “These decisions happen very quickly, so where we’re saying team decisions, I’m talking like 20, 30 seconds of discussion and then we’re done, and that’s normally discussion whilst I’m putting a wire into the femoral artery, so the procedure’s already starting.” S03L0091, Registrar.  “So the decision making is happening in a space and time dimension that’s changing and then you’re (inaudible - audio interference) in a position where you’re at hospital now and you’re in the process still of making the decision.” S03L0109, Consultant. |
| **4**  **Beliefs about capabilities**  Perceptions about her/his own competence/self-confidence in performing REBOA and recruiting to PPRO.  Self-efficacy – a person’s confidence that s/he can employ the skills that are necessary to enact REBOA and recruit to PPRO.  Domain frequency: 12/14 | I am (not) confident about delivering REBOA^2^ | 16/9 | “I think people are becoming more comfortable about REBOA generally with more training.” S03L0091, Registrar.  “I think with PPRO it will be concerning because it will be more often two-person teams, and I think the ability to optimally deploy zone one REBOA in a two-person team, I mean, I find that intimidating, there’s so much to do.” S03L0101, Consultant. |
|  | Determining eligibility for PPRO is difficult^12^ | 7/5 | “I feel fairly confident in recruiting patients to the P-PRO. I suppose the challenge for us is which zone it goes in. Part of it is people are pretty good at looking at whether something is disruptive, but whether somebody’s bleeding from their abdomen is really difficult.” S03L0096, Paramedic.  “It was very clear: these are the inclusion criteria, these are the exclusion criteria. If it’s a yes, do this, if it’s a no, do this. You know that sort of thing. From that point of view, I think I am sufficiently comfortable with the process of enrolling someone into the research study with the education that [name] and the team’s given us.” S03L0107, Paramedic. |
| **5**  **Social influences**  External pressure from other people/ views of other professions/ patients that influence healthcare professional behaviour about recruiting to PPRO and deploying REBOA.  Domain frequency: 13/14 | Our team is enthusiastic about REBOA^2^ | 9/7 | “The overall bias is that REBOA is a very positive thing in this proportion of patients, but the emphasis of getting it right and delivering it to the right patient is extremely high, the emphasis of making sure that your thought process is extremely clear, the team feels that this is the right thing to do because of the whole patient package, the scene, the mechanism, reading the patient assessment, what we believe is happening.” S03L0093, Paramedic.  “Everyone who is doing it is so enthusiastic about it, whether they are all ‘Great job guys, well done.’ I am not ever sure whether someone would say ‘Do you know what this really wasn’t the right patient to recruit and why on earth did you do it?’ We need to look for some more dissenters I think.” S03L0095, Consultant. |
|  | Team dynamics influence recruitment and intervention delivery^12^ | 7/7 | “Yes, so all the decisions are made by the team together. When we attend the patient, it’s a team effort of assessment, critical thinking and coming up with a management structure.” S03L0093, Paramedic.  “But then the next step is the procedure and again that’s relatively straightforward, but the politics and the challenges of taking that training into an environment I’m not familiar with – which is the theatre environment – alongside the surgeons who I’ve already said are not exactly the most receptive to this procedure and it’s very variable as to who might be on… and we don’t know them, you know? I don’t work alongside surgeons. I know the registrars but I don’t work alongside the consultant surgeons. That lack of familiarity with the surgical teams is a bit more of a challenge. I mean, the anaesthetists tend to be a lot better with this, but even they… you know, there’s a high degree of scepticism with that because they have to manage the patient after the balloon comes down and stuff.” S03L0103, Consultant. |
|  | Some clinicians lack equipoise^12^ | 7/5 | “The only issue I think will come when they don’t feel there’s that balance of equipoise. There’s passionate people who are very pro and people who aren’t, but I suppose the thing is that the vast majority of clinicians who are involved in this also understand the importance to the population of these patients to answer this question.” S03L0103, Consultant.  “But the thing that might be difficult for some clinicians is if they really think the patient in front of them would benefit from a Zone I balloon and they’re told not to do it, and that might be... I don’t know. But I personally wouldn’t have an issue with that, because we don’t know whether there’s clinical benefits to those patients, so from a research point of view, I’d be totally comfortable with it and not putting one in. But I think some of my colleagues might not necessarily be if they’d got a young 21 year old who’s dying in front of them and they’re unable to do it, but they’ve got a balloon that might bridge them ten minutes to hospital, it might... it might throw up some interesting ethical discussions and it would be interesting to see how many crossovers happen” S03L0091, Registrar. |
| **6**  **Behavioural regulation**  Ways of doing things that relate to pursuing and achieving desired goals, standards or targets. Personal strategies used to facilitate PPRO recruitment and REBOA intervention delivery.  Domain frequency: 12/14 | I use memory aides to help me recruit to PPRO and deploy REBOA^2^ | 7/4 | “The other thing that’s quite useful is in the REBOA equipment bags themselves have got aide memoires and cards into... for cues for the crews to make sure they remember the key bits of information around distances and balloon volumes and all that sort of stuff…so that’s all in that as an aide-memoire so I guess that would fall into this remit as well.” S03L0091, Registrar.  “Then, obviously, the technical aspects of performing successful REBOA, the total or partial… in our REBOA packs, at the back there is a troubleshooting guide along with some clear, very concise information to help you with the insertion depth and inflation, etc.” S03L0093, Paramedic. |
|  | I vocalise my actions to create structure during PPRO recruitment^12^ | 4/3 | “The other thing that I tend to do is I am very vocal obviously, so I tend to describe and articulate my thought process for the team, so it’s a shared decision making and then I overtly have a stop moment. Even if people are trying to die in front of me, I’ll have a little time out and make sure that we’re all on the same page and then we push on. I guess that’s a personal way that I do this job, and I guess that’s helpful for me because then that structure... and it’s help for me because it structures every job the same: I go to a patient, I hear what’s happened, I examine the patient, I talk through my examination, and I come up with what I think’s going on and I say, “This is what we’re going to do.” And then we have a conversation and then we do it.” S03l0091, Registrar.  “But the things that we do to try and bring any scene under control are the same that we do whether it’s a PPRO or not, which are all about communication, primacy of care, dissemination of plan, role allocation, you know?” S03L0101, Consultant. |
|  | I prepare my kit for PPRO^2^ | 3/3 | “There are a few things, one is the preparation so your kit is prepared and your team is prepared to the point where the things you don’t need to do when you get to scene are already done. Their either done by preparing your kit and being confident that it is what it is when you open the bag.” S03l0095, Consultant.  “The strategy for me, in my head, is know the kit inside out – literally with my eyes closed – understand exactly what the procedure is and how I can help troubleshoot it because if one of the regs or one of the consultants is having a bad day and something just isn’t working, then understanding that.” S03L0107, Paramedic. |
| **7**  **Goals**  Priorities, importance, commitment to a certain course of actions or behaviours (PPRO recruitment)  Domain frequency: 13/14 | We have goals and targets to meet in PPRO^1^ | 10/10 | “I think if I’m right the feasibility study needs eight patients, that’s my understanding.” S03L0098  “In my head I don’t think there’s any quotas for recruitment. We… it’s just the indications I suppose, there’s no quotas as far as I’m aware.” S03L0096 |
|  | I find goals and targets for recruitment unhelpful^1^ | 10/9 | “It’s difficult if you are cited on them and you’re actively looking to recruit patients and you risk being biased. The risk of bias is just the thing of recruiting patients that didn’t need it and then they have relatively good outcomes and then it makes like it is the right thing to do. It’s difficult as it comes back to that individual judgement a little bit.” S03L0095, Consultant.  “But yeah, it needs to be left to the investigators, but for people on the day to day, I think the pressure of recruiting a number shouldn’t have any influence on the decision-making process and there’s a problem if it does. I’m not sure entirely how helpful they are.” S03L0101, Consultant. |
| **8**  **Skills**  Competence, ability required/proficiency acquired through practice to recruit patients to PPRO and deploy REBOA.  Domain frequency: 12/14 | REBOA requires specialist technical skills^2^ | 13/9 | “There is an element of it being technically difficult and one of my colleagues previously has described canulating the common femoral artery as canulating a pixel obviously the... because sometimes it’s like one or two millimetres so it’s tiny, so it is technically challenging, and you do need to have practiced that.” S03L0091, Registrar.  “The core skill of being able to gain vascular access in the patient that is incredibly flat, that is incredibly... is generally unstable and that, again, we’re lucky that we’ve got experienced clinicians that would do that, so it’s not... from our point of view, the paramedic group do not do vascular access from a... you know, going into their groin, that’s not something we do; that is left to the doctors on the team.” S03L0107, Paramedic. |
|  | Decision making in prehospital trauma requires specific expertise^12^ | 12/7 | “There’s definitely an experience of understanding what clinically unsurvivable injuries are, and unfortunately, within [service]…we have a lot of exposure to those individuals that are traumatically injured with horrific injury load that they are unsurvivable, so that’s definitely a skill, I suppose.” S03L0107, Paramedic.  “You need to have enough experience, I suppose so you’ve got the intuition as to who is actually sick enough to warrant the intervention. The intervention is not without its potential dangers, and therefore you need to be able to pick up the ones that are going to benefit, and you need to be able to do so quickly, really.” S03L0108, Consultant. |
|  | Communication skills are critical in fast-paced prehospital environments^12^ | 4/3 | “The key thing is communication, for me, and it always comes down to communication and that be... whether it’s communicating around what we’ve found on primary survey, what the plan is going to be, what the procedure is, what we’re going to undertake…” S03L0092, Paramedic.  “Scene safety… it all comes down to… the unique skill set of pre-hospital care is just communication, you know? There’s no technical skill. And the ability to have been in enough scenes that you’ve got bandwidth to deploy that communication, and actually that’s not unique to REBOA or the PPRO scenes.” S03L0101, Consultant. |
|  | Maintaining competency can be challenging due to scarcity of cases^2^ | 3/2 | “The cases are so few and, you know, there’s a fair number of clinicians that need to be able to do it if the patient needs it and you’re only doing a handful of cases per year. Keeping those skill sets up and decision-making up is difficult.” S03L0111, Surgeon.  “I guess doing the REBOA is a technical... it’s a technical skill, right, so you need to be able to be technically good at what you’re doing, but the only real technicality you need to be good at is instrumenting the common femoral artery with an ultrasound machine, which most doctors that have worked in critical care or emergency medicine, can do. We just don’t normally see patients in the level of extremist that we see them in, so they’re the sickest of the sick, so it is hard.” S03L0091, Registrar. |
| **9**  **Emotion**  Feelings or affect about conducting REBOA and recruiting to PPRO.  Domain frequency: 11/14 | Emotions affect me only after recruitment and intervention delivery^12^ | 4/4 | “I don’t think emotions on scene for most of us get that involved. It’s only afterwards when we go, “Oh, shit, that was a sad one” or “That was bad” and then we allow ourselves to be emotive at that point. I don’t allow myself during a job to get emotive because then the patient care will suffer.” S03L0091, Registrar.  “I think they [emotions] probably come into play after the fact. I think at the time, there isn’t really scope or space in your brain to be considering feelings and emotions. It’s very much more a task intervention relationship transaction, really. I don’t ever really remember from any job with a critically ill patient thinking about emotion. I mean, potentially once you have done your intervention and removed to hospital, they may creep in, and they certainly will after the fact.” S03L0108, Consultant. |
|  | Feelings and emotions don't affect me in the workplace^12^ | 4/3 | “No, I’m not a particularly emotional person at work, if I’m honest with you. I’m quite didactic in approaching things, and from an analytical point of view, we’re being provided with a set of criteria, inclusion exclusion, this person needs to tick this box.” S03L0107, Paramedic.  “Yes, I guess... the older the patient, I guess, there’s probably, in my mind, there’s maybe a few more risks associated with that. But emotionally, I don’t think so. I don’t think there’s... I don’t think I feel particularly... I don’t know, different.” S03L0092, Paramedic. |
|  | I worry about conducting REBOA and managing the situation^2^ | 3/3 | “I would feel worried about REBOA being the wrong decision for the patient. I worry about the zone being the wrong decision. I worry about not getting the balloon in the right vessel. Those are the things I worry about. I worry about being unsuccessful with the procedure and delaying their care to the point where I would look back and wished I hadn’t tried to do it.” S03L0095, Consultant.  “It is really hard to make a decision when there’s so much uncertainty and you’re going to embark on a really complex procedure that’s going to take time that the patient doesn’t have, in the hope that it’s beneficial. I think that definitely does play on my mind…” S03L0111, Surgeon. |
| **10**  **Optimism**  The confidence expressed that things would happen for the best (e.g. running an RCT of REBOA in the prehospital environment will be successful).  Domain frequency: 8/14 | I am optimistic about doing an RCT of PPRO^12^ | 8/7 | “I think the elements of the trial so far in hospital suggest to me that actually the logistics of it in pre-hospital should be more straightforward with dedicated teams who understand the trial.” S03L0103, Consultant.  “Do I think we could do a trial where we pick a number of patients and randomise them to one thing or another? I think, yes if we got over the equipoise issue, yes, I think we could successfully deliver that trial.” S03L0108, Consultant. |
|  | An RCT of PPRO will be challenging^12^ | 7/6 | “I guess my concerns with that are twofold. One is that currently that would be a single centre study because there’s nowhere else doing it. My other one if we look at the number of P-PRO patients there’s been, which is so small, then to do an RCT that’s appropriately powered is going to take decades.” S03L0091, Registrar.  “I think the fundamental problem with an RCT, as with the trials I’m dealing with are the small numbers, and it’s very difficult to then take anything in terms of outcome benefits statistically as meaningless.” S03L0101, Consultant. |
| **11**  **Intentions**  A conscious decision to perform a behaviour, and stability of intentions.  Domain frequency: 8/14 | Patient care comes first ahead of any recruitment^1^ | 7/6 | “For me, I’m pretty clear that... you might not want to hear this, but things like studies and things like that are always second for me, so the first thing for me is the patient and their dignity and make sure that they are absolutely getting the care and the initial care they need and that is normally volume resuscitation and a bit of dignity and algesia. I never take a patient to hospital in pain.” S03L0091, Registrar.  “I don’t know if I’m motivated to recruit someone. I think it’s just trying to do the right thing for the right patient at the time.” S03L0092, Paramedic. |
|  | I want to establish the effectiveness of REBOA^2^ | 1/1 | “But that’s still significant, so all those blunt patients need another intervention and therefore this... my other personal motivation is to work out what that intervention is for these patients because some of them should... some of them have salvageable injuries on post-mortem. Some of them obviously don’t and they’re catastrophic at the time of impact but some do, and therefore that’s my other thing. It comes back to just wanting to help more people…” S03L0091, Registrar. |
| **12**  **Knowledge**  Existing procedural knowledge, knowledge about REBOA and PPRO (e.g. patient eligibility, evidence for/against REBOA).  Domain frequency: 6/14 | I am aware of the eligibility criteria^1^ | 5/5 | “I think the advantage you have with REBOA is that most people know the criteria already, it’s not a new drug that has a very specific inclusion criteria and people are familiar with the technique and the patient cohort.” S03L0101, Consultant.  “But, yes, there’s very clear sets... inclusion criteria and then very clear set exclusion criteria, which are in the study protocol, so we would run through those... you know, adult trauma patient above the age of sixteen, must have subdiaphragmatic exsanguinating haemorrhage that the REBOA would work and then it’s a bit about advance pregnancy and that sort of thing.” S03L0107, Paramedic. |
|  | I am aware of the evidence base for REBOA^2^ | 2/1 | “The second thing is there is lots of really good evidence and there’s lots of logic to putting a balloon up and doing partial REBOA.” S03L0095, Consultant. |
| **13**  **Social professional role and identity**  Expressions about their own professional identity/job/role/professional boundaries and emotion comparisons about their role with that of other professionals. How their professional roles/identities influence recruitment to PPRO and delivery of REBOA.  Domain frequency: 7/14 | Decision making is determined by professional roles^12^ | 7/4 | “When there’s a consultant on the team then the decision making although it amongst all of us, I guess, defaults to them as they are the person that’s responsible ultimately for the patient’s care.” S03L0091, Registrar.  “And then it’s running the wider patients, so those tasks are fairly task-focussed, so we call it “eyes-in”, so the doctors are very task-focussed and eyes-in, so part of my role is to make sure that the patient is receiving all of the other supported measures that are required.” S03L0093, Paramedic. |
|  | Capability to perform REBOA depends on professional background^2^ | 5/3 | “[confidence is] Pretty high, just because my job that’s not [service] is as a consultant, so I’m used to putting cannulas into vessels and groins all the time. The technical aspects don’t cause me stress. Having done a lot of cannulation, I’m also very aware that there are patients that can’t be cannulated so I don’t get myself in a stress if it will be difficult to get access or not possible to get access.” S03L0101, Consultant.  “For some of the doctor cohort, again, I can’t speak specifically for them, but some of the skills of getting arterial access and things like that are things that they’ve done in their general practice throughout their time, so it’s not familiar although doing it pre-hospital is unfamiliar.” S03L0093, Paramedic. |
| **14**  **Reinforcement**  Whether any financial/non-financial incentives/ positive or negative consequents influence healthcare professional behaviour & decisions to recruit to PPRO/deploy REBOA.  Domain frequency:  7/14 | Goals and targets might bias patient recruitment^1^ | 8/7 | “It’s difficult if you are cited on them and you’re actively looking to recruit patients and you risk being biased. The risk of bias is just the thing of recruiting patients that didn’t need it and then they have relatively good outcomes and then it makes like it is the right thing to do.” S03L0095, Consultant.  “Whilst research is important, I don’t think that we would inappropriately enrol someone or not. No, I think the group of clinicians are too patient-orientated from that point of view, rather than doing something that was naughty for the purposes of research.” S03L0107, Paramedic. |

1. Applies to recruitment [↑](#footnote-ref-1)
2. Applies to intervention delivery [↑](#footnote-ref-2)
